# Impact of exercise programs among helicopter pilots with transient LBP

**DOI:** 10.1186/s12891-017-1631-0

**Published:** 2017-06-20

**Authors:** Knut Andersen, Roald Baardsen, Ingvild Dalen, Jan Petter Larsen

**Affiliations:** 1 Department of Research, Stavanger University Hospital and Network for Medical Sciences, University of Stavanger, PB 8100, , N-4068 Stavanger, Norway; 2 Department of Neurosurgery, Stavanger University Hospital, PB 8100, , N-4068 Stavanger, Norway; 3 Department of Research, Section of Biostatistics, Stavanger University Hospital, PB 8100, , N-4068 Stavanger, Norway; 4 Network for Medical Sciences, University of Stavanger, Postboks 8600 Forus, 4036 Stavanger, Norway

**Keywords:** Low back pain, Lumbar trunk, Muscular endurance, Exercise, Helicopter pilots

## Abstract

**Background:**

Flight related low back pain (LBP) among helicopter pilots is frequent and may influence flight performance. Prolonged confined sitting during flights seems to weaken lumbar trunk (LT) muscles with associated secondary transient pain. Aim of the study was to investigate if structured training could improve muscular function and thus improve LBP related to flying.

**Methods:**

39 helicopter pilots (35 men and 4 women), who reported flying related LBP on at least 1 of 3 missions last month, were allocated to two training programs over a 3-month period. Program A consisted of 10 exercises recommended for general LBP. Program B consisted of 4 exercises designed specifically to improve LT muscular endurance. The pilots were examined before and after the training using questionnaires for pain, function, quality of health and tests of LT muscular endurance as well as ultrasound measurements of the contractility of the lumbar multifidus muscle (LMM).

**Results:**

Approximately half of the participants performed the training per-protocol. Participants in this subset group had comparable baseline characteristics as the total study sample. Pre and post analysis of all pilots included, showed participants had marked improvement in endurance and contractility of the LMM following training. Similarly, participants had improvement in function and quality of health. Participants in program B had significant improvement in pain, function and quality of health.

**Conclusions:**

This study indicates that participants who performed a three months exercise program had improved muscle endurance at the end of the program. The helicopter pilots also experienced improved function and quality of health.

**Trial registration:**

Identifier: NCT01788111 Registration date; February 5th, 2013, verified April 2016.

**Electronic supplementary material:**

The online version of this article (doi:10.1186/s12891-017-1631-0) contains supplementary material, which is available to authorized users.

## Background

Low back pain (LBP) among helicopter pilots has been described since the 1960’s [1]. The literature describes two distinct pain patterns of LBP among pilots [1, 2]; transient LBP related to flying that ceases shortly or within hours after a flying mission, and a more recurrent chronic pain pattern not necessarily related to flying and possibly present before and/or after flying [2].

The transient LBP pattern has been attributed to flying time, vibration, types of aircraft, ergonomics of the cockpit and seat design with lack of regulation possibilities of the cockpit seat. Anthropometrical factors of the pilots have also been considered as well as the role of body mounted gear for safety. In addition, the pilots’ own ability to endure the strain of prolonged confined sitting has received increased attention. Especially strategies to increase the pilots’ muscular ability to withstand the occupational distress related to piloting have been suggested.

Sitting is not, on its own, associated with LBP but prolonged awkward and constrained sitting show an increase in risk of LBP [2–5]. Static posture is tolerable, however, over longer periods movement and changes in sitting angles are necessary to provide periodic rest and optimal physiology of the muscles [5, 6]. Flight related lumbar pain can therefore be caused by posture and muscular fatigue due to extended periods of static positioning [1, 2, 7]. Fatigue and pain are generally alleviated by adjustments of the sitting position, however opportunities for this is restricted in a helicopter seat due to harnesses and body mounted safety gear.

The lumbar trunk musculature works synergistically with the spine, pelvis, fascia and ligaments to stabilize the lumbar spine [8]. The active muscular structures are responsible for motion of the lumbar trunk in multiple directions [8]. Loss of strength and endurance of these muscles is considered predictive for the development of LBP [6, 9]. The extensors of the lumbar trunk; iliocostalis lumborum, longissimus thoracis and the deep and superficial portion of the lumbar multifidus muscle, (LMM) have been extensively studied. In relation to patients with LBP diagnostic ultrasound has been used to evaluate the LMM for size and contraction [10].

Several studies describe exercise and training programs for military helicopter pilots [11–14]. Some studies focus on outcomes such as pain or function [13, 14]. Others focus on lumbar trunk muscular endurance [12, 14, 15]. Still, there is a need for studies to evaluate in detail the impact of training programs on transient LBP among helicopter pilots.

The aim of this study was to investigate if muscular function improves after three months of an exercise program among commercial helicopter pilots reporting flying related transient LBP.

## Methods

### Study design

This is a prospective study of the impact of exercise programs on commercial helicopter pilots with transient LBP related to flying. The primary outcome was to measure changes in lumbar trunk muscular endurance. Secondary outcomes were alterations in isometric contraction of the LMM as well as changes in pain, function and quality of health before and after 3 months of intervention.

### Subjects

**Fig. 1:**
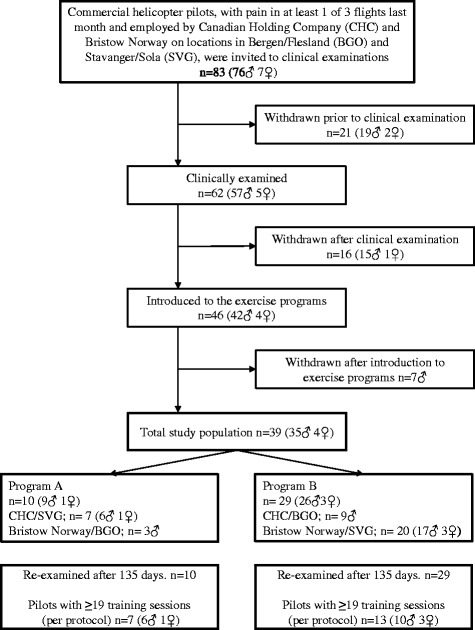
Flowchart

In a retrospective study from 2013 we found that 50% among commercial helicopter pilots (*n* = 207) reported transient LBP related to flying defined as LBP on at least one of three flights last month [16]. These pilots were all employed full-time by the two largest commercial helicopter companies in Norway, Canadian Holding Company (CHC) and Bristow Norway and served on several bases along the Norwegian coastline. The pilots operated Sikorsky S92, Eurocopter AS 332 L/L1 Super Puma and Eurocopter EC225 Super Puma. For practical and logistical reasons, only pilots with transient LBP from Bergen (BGO/Flesland) and Stavanger (SVG/Sola), 83 in total (76 men and 7 women), were eligible for the study (see flowchart, Fig. 1). Twenty-one pilots withdrew prior to the clinical examination because of pregnancy, foreign residency, moving to another location, sick leave for conditions other than LBP or no desire for further participation. For similar reasons a further 16 pilots withdrew after the initial examination, but prior to allocation and introduction to the training programs. In addition, seven male pilots withdrew after introduction to the exercise programs based on time constraints and difficulty in combining work, family and social life with time-consuming training sessions. Inclusion criteria for participation in this study was pilots that in the retrospective survey reported transient LBP related to flying on at least one of 3 flights last month. Further they had to be located at the bases in Stavanger and Bergen. As medically licensed and full-time operative pilots none of the pilots met any health-related exclusion criteria for participation. The study sample consisted of 39 pilots (35 men and 4 women). The recruitment period with initial clinical examinations, training and follow-up ran from October 2013 through August 2014 for the Stavanger group of pilots and from February 2014 through December 2015 for the Bergen group. The Western Norway Regional Committee for Medical and Health Research Ethics approved the study protocol (REK Vest 2010/2254).

### Examinations

The initial examination comprised questions regarding past injuries, treatments and conditions that might affect their ability to train. Standard low back orthopedic and neurological examination as well as range of motion assessment of the lumbar trunk were also done. The pilots completed questionnaires both at the initial examination and after the intervention period. The questionnaires included measurements of the average intensity of the LBP last week using a Visual Analog Scale (VAS; 0–10), impact of LBP on activities of daily living using the Oswestry Disability index (ODI; 0–100), and the EQ-5D Health-related quality of life questionnaire (a profile with a single index, 0–100, for health status).

Participants were tested for lumbar trunk muscular endurance, without tactile or verbal feedback, in extension, flexion and side-bridge on both sides with values given in seconds [6] (see Additional file 1: Appendix 1). The back extensors were tested in a Biering-Sorensen position with the upper body cantilevered over the edge of the test bench and the ankles secured. For the flexion test we applied a 55 degrees angled jig with the feet anchored under the foot support, knees at 90 degrees and the upper torso resting towards the jig. The test started when the jig was pulled back 10 cm. The side bridge tests were performed on either side with elbow support, legs fully extended with the top foot in front of the lower and the body in a straight line. Before starting each test, identical verbal and visual instructions were given to all participants. Similar testing procedure has been validated through reliability studies with Intraclass Correlation Coefficient (ICC; 0.00 to 1.00) at 0.66 for trunk flexion, 0.79 for trunk extension and 0.74 and 0.96 for right and left side bridge respectively [17]. The tests were at random conducted by a physiotherapist or the main author after procedural agreement including determination of endpoints.

Isometric contraction of the LMM was assessed using real-time ultrasound scanning. This was done bilaterally on a longitudinal view at the level of the facet joints of L4-L5 and L5-S1. In a prone position, the candidate lifted the opposite upper extremity of the side being measured. The difference in muscle thickness between relaxed and isometrically contracted LMM was measured in mm with change given in percent [18]. Similar examination procedure has been validated through reliability studies with an intraexaminer design, comparable to the present study, at 0.97 for measurements of the LMM in a resting state and 0.99 in the contracted state. The ICC value for the change between relaxed and contracted LMM was 0.78 [19]. We used Samsung Medison Accuvix XG, 3366 Hanseo-ro, South Korea for the Stavanger group and Medison SonoAce 8000 EX, 3366 Hanseo-ro, South Korea for the Bergen group, using a 3–7 MHz curvilinear probe on both machines.

### Intervention

Several exercise programs have been reported to increase LT muscular endurance. We included both a traditional program (A) and a more specific program (B) designed to increase LT muscular endurance (see Additional file 2: Appendix 2A and Additional file 3: Appendix 2B). Program A consisted of 10 exercises each performed in series of 3 × 10 repetitions. The exercises used in Program A are generally accepted among health professionals as addressing low back complaints. All pilots allocated to program A had an instruction class led by a physiotherapist with demonstration of the exercises. There were no specific instructions for the execution of the exercises.

Program B consisted of 4 exercises to be performed in the same sequence in all exercise sessions. The exercises were explained and demonstrated either on an individual level or in small groups (maximum 3 pilots) led by the main author. Three exercises were performed on an “angle training bench” and one “curl-up” exercise on a floor mat. All pilots were instructed and rehearsed in “bracing” [6]. “Bracing” should be initiated and released, with decreasing intensity upon increasing skill, between each repetition. Every repetition comprised an initial steady fast concentric phase, followed by an isometric phase and a closing eccentric phase. All exercises were performed in 4 series according to a “reversed pyramid” protocol [6]. In general, the series were separated by one-minute intervals involving brisk walking or cycling a stationary spinning bicycle. All exercises were performed according to the principle of progressive overload [20].

The equipment and training facilities were provided by the companies and made available at the bases in Bergen and Stavanger. During the training period participants of both programs received encouragement via email from both the instructing physiotherapist and the main author.

The 39 pilots included in this study were allocated to two training programs (A and B) depending on company affiliation and location. CHC pilots performed program A in Stavanger and program B in Bergen. Pilots from Bristow Norway had an opposite distribution. A total of 10 pilots (9 men and 1 woman) trained program A and 29 pilots (26 men and 3 women) program B (see flowchart Fig. 1). The intervention period was scheduled to end after 135 days and/or 36 training sessions. All participants were issued a training diary for recording their sessions with number of repetitions performed.

### Statistics

Descriptive statistics are presented as medians and interquartile ranges (IQRs) or as means and standard deviations (SDs) for all pilots included. In addition, for male pilots as well as for the subset of pilots included in the per protocol analysis performing 19 or more training sessions; i.e. more than 50% of the scheduled 36 training sessions. Pre-training and post-training scores were compared using paired samples T-test, and presented results include estimated mean differences with normal based 95% confidence intervals (CIs). Supplementary, non-parametric tests (Wilcoxon) were performed to validate the findings. Observed differences with *p* < 0.05 were considered statistically significant. SPSS v.23 was used for all statistical analysis.

## Results

Of the 39 pilots included in this study population, 20 pilots performed per protocol regardless of program. Of the 10 pilots included in Program A, 7 pilots (70%) performed per protocol and had a median number of 35 training sessions. The median number of training sessions for all 10 pilots was 29.5. The median number of training sessions for all 29 participants in program B was 17. The subset of 13 pilots (45%) in program B, who performed per protocol had a median of 27 training sessions.

Table 1 shows the baseline demographic characteristics of the participants in the training programs. There were no substantial baseline differences in the demographic features between the total study group of 39 pilots or the subset population of 20 pilots who performed per protocol. Neither were there any substantial differences in the baseline demographic characteristics of the male pilots per se or among the participants in program A and B.

**Table 1:** Baseline characteristics of all included helicopter pilots irrespective of training program. Values in median and Interquartile range (IQR)

	All included *n* = 39	Male pilots *n* = 35	Per protocol^1^ *n* = 20
Sex	35 males (90%)	35 males (100%)	16 males (80%)
Age years	36 (33, 43)	37 (33, 44)	36.5 (34.3, 42.8)
Height cm	183 (176, 185)	183 (178, 187)	181.5 (173.8, 188.5)
Weight kg	85 (78, 93)	86 (78, 94)	85.5 (79.3, 94.8)
BMI	25.7 (24.3, 27.1)	25.7 (24.3, 27.1)	26.3 (24.6, 27.6)
VAS (0–10)	3 (2, 5)	3 (2, 5)	3 (2, 5.8)
ODI %	10 (6, 14)	10 (6, 14)	9 (6, 13.5)
EQ-5D (0–100)	79 (70, 88)	79 (70, 88)	79 (73.5, 90)
Flying experience			
Years	12 (8, 16)	12 (8, 17)	11 (8.3, 16.8)
Flying hours	4000 (3400, 7000)	5000 (3500, 7000)	4400 (3330, 6760)
Hours in “Large” helicopter	2900 (2100, 4800)	3200 (2100, 4800)	3150 (2100, 4800)
Hours last year	600 (450, 700)	620 (460, 700)	600 (463, 700)
SVG^2^	30 (28 males)	26 (males)	16 (12 males)
BGO^3^	9 (males)	9 (males)	4 (males)
S92^4^	32	29	16
AS332 L/L2^5^	7	6	4
EC225^6^	-	-	-
Muscular endurance in seconds			
Extension	47 (30, 78)	47 (30, 78)	59 (34, 83)
Flexion	55 (38, 83)	55 (38, 83)	45 (34, 82)
RSB^7^	32 (24, 46)	33 (25, 47)	31 (23, 44)
LSB^8^	39 (20, 52)	40 (26, 52)	44 (31, 57)
Total holding time	190 (140, 253)	196 (140, 253)	199 (142, 268)
Ultrasound in percent^9^			
Level L4–5	8.1 (5.2, 11.1)	8.3 (5.7, 11.1)	7.3 (5.1, 10.3)
Level L5-S1	6.3 (2.1, 8.6)	6.6 (2.1, 8.6)	4.1 (2.1, 7.2)
Right side L4–5 & L5-S1	5.9 (2.5, 9.5)	6.4 (3.3, 9.5)	4.5 (2.3, 9.1)
Left side L4–5 & L5-S1	6.8 (3.7, 12.4)	6.8 (4.0, 12.4)	6.4 (3.7, 10.2)
Both levels and sides	7.5 (3.7, 9.7)	7.7 (3.9, 9.7)	5.3 (3.6, 8.0)

^1^Per protocol: pilots performing ≥19 of scheduled 36 training sessions. ^2^SVG = Stavanger/Sola base. ^3^BGO = Bergen/Flesland base. ^4^Sikorsky helicopter type S92. ^5^Airbus (Eurocopter) Helicopter Super Puma type AS 332 L/L1/L2. ^6^Eurocopter EC225 Super Puma (Airbus Helicopters H225). ^7^RSB = Right Side Bridge. ^8^LSB = Left Side Bridge. ^9^Values given in %-change from relaxed to contracted muscle

### Outcome and measures before and after intervention

Table 2 shows the clinical features and test results of all 39 pilots and of the 20 pilots who performed per protocol before and after the intervention. All the pilots, including those who performed per protocol, regardless of program, demonstrated marked improvements in muscular endurance. The mean total holding time increased from 198 s to 298 s (*p* = 0.001). Improvement in the contractility of the LMM at both lumbar levels L4–5 and L5-S1 as well as and in combination of lumbar levels and sides (*p* = 0.000). There was significant improvement in function (ODI; *p* = 0.010) and in health-related quality of life (EQ-5D; *p* = 0.001) after training. General LBP was not significantly changed (VAS; *p* = 0.069). Non-parametric tests showed similar results.

**Table 2:** Pre- and post-training values^1^ of all included pilots along with the subset of pilots performing per protocol

	All included pilots *n* = 39				Pilots with ≥19 training sessions, per protocol *n* = 20			
	PRE	POST			PRE	POST		
	Mean (SD)	Mean (SD)	Mean diff (95% CI)	*p*-value	Mean (SD)	Mean (SD)	Mean diff (95% CI)	*p*-value
VAS (0–10)	3.5 (2.0)	2.3 (1.6)	÷1.2 (÷2.0 to ÷0.4)	.005	3.5 (2.2)	2.3 (1.5)	÷1.3 (÷2.6 to 0.1)	.069
ODI %	10.5 (6.2)	7.1 (6.0)	÷3.3 (÷5.0 to ÷1.6)	.000	10.1 (5.6)	7.7 (5.2)	÷2.4 (÷4.2 ÷0.6)	.010
EQ-5D (0–100)	82.3 (13.1)	89.6 (12.4)	7.4 (2.9 to 11.9)	.002	77.7 (13.1)	90.2 (12.8)	12.5 (5.9 to 19.1)	.001
Muscular endurance in seconds								
Extension	54 (28)	56 (29)	2.7 (÷8.4 to 13.9)	.622	57 (28)	63 (36)	6.3 (÷13.8 to 26.4)	.523
Flexion	65 (37)	111 (54)	45.8 (29.0 to 62.6)	.000	62 (40)	109 (56)	47.4 (18.6 to 76.2)	.003
RSB^2^	34 (16)	52 (20)	17.9 (12.1 to 23.7)	.000	35 (18)	52 (20)	17.7 (9.7 to 25.6)	.000
LSB^3^	40 (20)	63 (29)	23.2 (15.7 to 30.7)	.000	45 (22)	73 (33)	28.3 (18.0 to 38.6)	.000
Total holding time	193 (70)	283 (98)	89.6 (59.5 to 119.8)	.000	198 (75)	298 (111)	99.6 (46.8 to 152.5)	.001
Ultrasound in percent^4^								
Level L4-L5	9.0 (6.1)	11.6 (5.9)	2.6 (1.1 to 4.1)	.002	8.0 (4.5)	10.4 (5.2)	2.4 (1.1 to 3.6)	.001
Level L5-S1	6.1 (4.6)	7.7 (4.8)	1.5 (0.4 to 2.7)	.011	4.7 (3.7)	6.5 (4.3)	1.8 (0.5 to 3.1)	.010
Right side L4–5 & L5-S1	6.8 (5.6)	9.4 (5.0)	2.7 (1.2 to 4.2)	.001	5.5 (4.3)	8.7 (5.0)	3.2 (2.1 to 4.2)	.000
Left side L4–5 & L5-S1	8.4 (5.7)	9.8 (6.3)	1.4 (0.0 to 2.8)	.044	7.2 (4.4)	8.1 (4.7)	1.0 (÷0.7 to 2.6)	.231
Both levels and sides	7.6 (5.1)	9.6 (5.2)	2.1 (0.8 to 3.3)	.002	6.3 (3.9)	8.4 (4.9)	2.1 (1.1 to 3.1)	.000

^1^Irrespective of training program. Mean values with standard Deviation and Mean difference with 95% Confidence Intervals (CI) and *p*-value form paired t-tests. ^2^RSB = Right Side Bridge. ^3^LSB = Left Side Bridge ^4^Values given in %-change from relaxed to contracted muscle

### Data from program A and B

Table 3 shows pre- and post-training measurements of pilots performing per protocol in program A and program B, respectively. Program A had statistically significant changes in endurance times in 3 of the 4 tested positions and significantly improved combined total endurance times in all 4 positions (*p* = 0.003). Participants in program A also increased contractility of the LMM in a total combination of sides and levels (*p* = 0.035). Program A pilots had no statistically significant improvements after intervention in function, pain or quality of life.

**Table 3:** Values for pilots performing per protocol^1^, program A and B respectively

	Program A per-protocol *n* = 7				Program B per-protocol *n* = 13			
	PRE Mean (SD)	POST Mean (SD)	Mean diff (95% CI)	p	PRE Means (SD)	POST Means (SD)	Mean diff (95% CI)	p
VAS (0–10)	2.4 (1.4)	2.9 (1.5)	0.4 (÷1.0 to 1.8)	.482	4.1 (2.4)	1.9 (1.4)	÷2.2 (÷4.0 to ÷0.3)	.028
ODI %	8.6 (5.9)	6.3 (5.1)	÷2.3 (÷6.6 to 2.0)	.244	10.9 (5.6)	8.5 (5.4)	÷2.5 (÷4.5 to ÷0.4)	.022
EQ-5D (0–100)	77.2 (17.6)	87.6 (12.0)	10.4 (÷5.0 to 25.9)	.150	78.0 (10.9)	91.6 (13.4)	13.7 (5.7 to 21.6)	.003
Muscular endurance in seconds								
Extension	67 (14)	95 (43)	28 (15.2 to 70.4)	.166	51 (33)	46 (14)	÷5.2 (÷28.2 to 17.7)	.628
Flexion	48 (18)	122 (49)	73 (26.1 to 120.8)	.009	69 (48)	102 (61)	33.4 (÷5.2 to 71.9)	.084
RSB^2^	41 (25)	60 (26)	19 (8.5 to 29.2)	.004	31 (11)	48 (16)	17.0 (5.1 to 28.9)	.009
LSB^3^	57 (28)	94 (42)	37 (19.0 to 54.1)	.002	38 (15)	62 (21)	23.9 (10.1 to 37.6)	.003
Total time holding time	214 (55)	371 (118)	156 (76.5 to 236.3)	.003	190 (85)	259 (88)	69.0 (÷1.4 to 139.4)	.054
Ultrasound in percent^4^								
Level L4-L5	6.9 (5.0)	9.1 (4.6)	2.3 (÷0.3 to 4.8)	.076	8.6 (4.3)	11.0 (5.5)	2.5 (0.8 to 4.1)	.007
Level L5-S1	5.1 (4.0)	5.9 (5.6)	0.9 (÷1.4 to 3.1)	.379	4.9 (3.7)	6.7 (3.7)	2.3 (0.5 to 4.0)	.016
ȀRight Side L4–5 and L5-S1	5.2 (4.6)	8.2 (5.2)	3.0 (0.9 to 5.0)	.012	5.7 (4.2)	9.0 (5.1)	3.3 (1.9 to 4.7)	.000
Left Side L4–5 and L5-S1	6.8 (4.4)	6.9 (4.6)	0.2 (÷2.1 to 2.4)	.878	7.4 (4.6)	8.8 (4.8)	1.4 (÷1.0 to 3.8)	.225
Both levels and sides	6.0 (4.2)	7.5 (4.8)	1.6 (0.2 to 3.0)	.035	6.5 (3.8)	8.9 (4.4)	2.4 (0.9 to 3.9)	.005

Mean values with Standard Deviation(SD) and Mean difference with 95% Confidence Intervals(CI) and *p*-values form paired samples t-test. ^1^Pilots performing per protocol ≥19 session of the scheduled 36 sessions. ^2^RSB = Right Side Bridge. ^3^LSB = Left Side Bridge ^4^Values given in %-change from relaxed to contracted muscle

Participants in program B achieved significant improvement in endurance in the side bridges.

They improved contractility of the LMM in a total combination of sides and levels (*p* = 0.005) as well as attained significant changes of contractility at both the measured levels at L4-L5 (*p* = 0.007) and L5-S1 (*p* = 0.016). Pilots in program B had significant reduction in pain (VAS; *p* = 0.028), and improvement in function (ODI; *p* = 0.022) and quality of life (EQ-5D; *p* = 0.003) after the intervention.

## Discussion

This study indicates that exercise programs increase muscular endurance in a sample of commercial helicopter pilots with flying related transient LBP. Both program A and B increased lumbar trunk muscular endurance and LMM contractility. Program B improved pain, function and health-related quality of life after training. Our findings indicate that helicopter pilots with transient LBP obtain marked improvement in function of the lumbar trunk muscles with training and that this is followed by less lumbar complaints related to flying.

The origins of LBP are heterogeneous and also controversial and most probably different mechanisms are involved in LBP in different individuals [21]. The importance of deconditioned lumbar trunk muscles has received increased attention in research and strategies for management of LBP [22]. Measurements of muscular endurance in the lumbar region and especially the ability to contract the LMM seem to be a valid measure of the function of the lumbar trunk muscles [23].

The hypothesis of specific lumbar extensor deconditioning as a causal factor in LBP is supported [9] by evidence suggesting that paraspinal muscles have significantly less contractility in patients with LBP than controls and significantly less contractility on the affected side in patients with unilateral LBP [10]. These factors along with other structural findings in the spine such as fat infiltration of the paraspinal musculature, decreased muscular quality and individual variations in muscular activation may contribute to recurrence of LBP [24]. Whether these structural changes are a cause or a consequence of LBP still remains elusive [25].

Lumbar trunk exercises aim to increase muscular strength and endurance. A meta-analysis from 2013 suggested that motor control exercises in patients with chronic or recurring LBP is superior to general exercises with regard to improved disability and pain [26]. However, a Cochrane review of motor control exercises for chronic non-specific LBP gave low to moderate evidence of pain reduction compared to minimal intervention [27].

Helicopter pilots with transient LBP experience increasing intensity of pain with longer sitting time. The prolonged static posture leads to muscular fatigue [2] and may represent a causal factor for flight related pain among pilots [2]. To our knowledge no studies have fully explored the impact of training on the endurance of the lumbar trunk muscles as well as possible improvement of pain and function in helicopter pilots. A study of five US Air force helicopter crew members did not evaluate lumbar trunk endurance but showed reduced inflight pain with no significant change in function (ODI) [13]. A Swiss study showed improvement in lumbar trunk endurance after training among army helicopter pilots without testing pain [12]. In the present study, we found a markedly longer muscular endurance and better contractility in LMM after the training period in the analysis of all included pilots. This was to some extent associated with better functioning and less pain. These findings thus support a relationship between dysfunctional lumbar trunk muscles and the complaints experienced by pilots. Moreover, this observation may also indicate that such malfunction may be involved in the LBP syndrome in general.

In addition, we included in this study two different training programs. Program B contained exercises that was designed to strengthen the lumbar trunk and a program A with general lumbar exercises. Both programs improved endurance and contractility of the LMM. The participants in program B also reported improved pain, function and quality of health. In program A we had too few participants to be able to evaluate any change in these features. Still, it seems important in future studies to clarify if a training program specially designed to strengthen the lumbar trunk muscles can provide a better outcome or not than a more general training program.

In studies with a longitudinal follow-up, there is always a risk of drop-outs among the participants**.** In this study, out of a total of 39 pilots, only 20 (51%) completed the study per protocol. There may be several reasons for this low compliance. The participating pilots had low to medium intensity of LBP and, therefore possibly limited motivation. The exercise programs were both time consuming and rather demanding, with exercises that should be performed to the level of submaximal exhaustion. Participants in program B also depended on specialized equipment, though made available at the company bases they did not have easy access to the specialized equipment in off-work periods. In future studies with similar objectives, it would be beneficial to increase the motivation for training by providing the opportunity to train within working hours and not as in this study on spare time [28].

This study has several limitations. First, the participants were examined pre and post training with a non-blinded study design. The lack of blinding could bias the measurements in this study of pre to post changes. Secondly, we experienced a high drop-out rate that left us with a rather low number of participants that had trained per protocol. Thirdly, there was no control group without training as the participating helicopter service companies objected to having a group of pilots with pain that would not be offered effective professional help for their ailments. Still, we found marked improvements in rather hard end-points like muscular endurance and LMM contractility after training. We therefore believe our findings are valid and they may therefore have consequences for management of transient LBP among commercial helicopter pilots. They should be encouraged to do such exercises and thus possibly reduce the development of chronicity of LBP, time at sick leave and quality of life.

## Conclusions

This study indicates that low back exercises improve endurance as well as the contractility of the LMM as a major contributor to lumbar trunk endurance. Further studies with a larger study populations with less attrition and with a RCT study design are needed to confirm these findings and to evaluate the effects of different training approaches and regimens among commercial helicopter pilots.

## Additional files

Additional file 1: Appendix 1. Title of data: Test of muscular endurance.(184 kb)

Additional file 2: Appendix 2A. Training program A. (6130 kb)

Additional file 3: Appendix 2B. Training program B. (395 kb)

## Abbreviations

BGO: Bergen
CHC: Canadian Holding Company
EQ-5D: Euroqol-5D
LBP: Low Back Pain
LMM: Lumbar Multifidus Muscle
LT: Lumbar Trunk
ODI: Oswestry Disability Index
REK (Norwegian): Regional Etisk Komite;
SVG: Stavanger
VAS: Visual Analog Scale; Regional Committee for Medical and Health Research Ethics
