# Supplementary material for: Impact of exercise programs among helicopter pilots with transient LBP
Source: BMC Musculoskelet Disord. 2017 Jun 20;18:269. doi: 10.1186/s12891-017-1631-0 (PMC5477756; doi:10.1186/s12891-017-1631-0)
Supplement: Supplementary file 1 — Title of data: Test of muscular endurance.(184 kb) [file 12891_2017_1631_MOESM1_ESM.pdf]

Appendix 1

Testing procedure for lumbar trunk muscular endurance [1]

Equipment: Angle bench, floor mat, 55° angled jig (equipped with anti-skid material on the bottom) and stop watch

General Instructions: This is to measure in seconds your lumbar trunk muscular endurance in 4 positions; extension, flexion and side-bridge on either side. The purpose is to measure how long you can maintain a steady posture in each position and the time starts once you are guided into the correct position and you say a clear and loud YES. You can stop whenever you like. You will not receive any verbal or tactile feedback during the test. If you deviate from the assigned position I will stop the test. You will be allowed some movement. (Intertester agreement allows 5 cm). There will be at least a 3 minute rest between the different tests.

Test 1 Extension

Bench settings: Horizontal foot piece. Chest piece lowered to 60° to give some support during positioning only.

The candidate is placed prone on the angle bench with feet securely fastened under the foot support. The body is placed with anterior superior iliac crest cantilevered on the superior edge of the tables foot piece, Hands and arms crossed on opposite shoulder in front of the chest. Candidate lifts the upper torso and is guided to a straight body line.

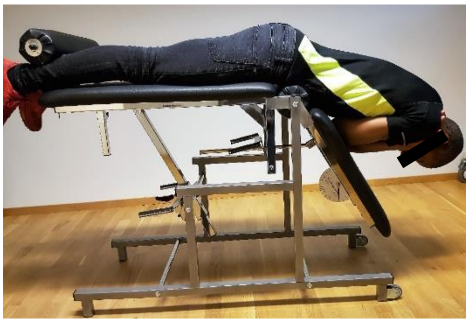

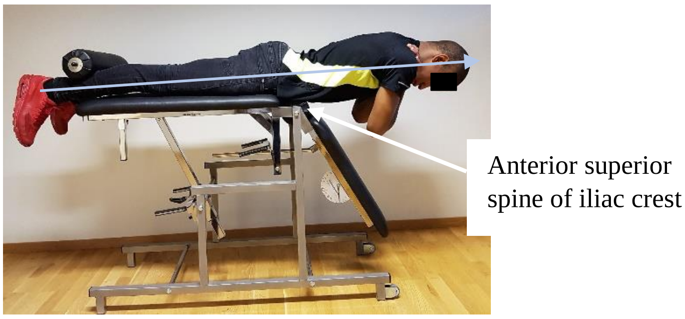

Test 2 Flexion

Bench settings: The entire bench is horizontal. The jig is put on top and position is adjusted to the length of the individual test person.

The candidate sit on top of the bench with feet firmly secured under the foot support with a knee angle of 90°. The upper torso is leaned against the 55° angled jig. The candidate is instructed to maintain that 55° angle according to general instructions as outlined above. The test starts when the jig is pulled back 10 cm.

Knees 90°

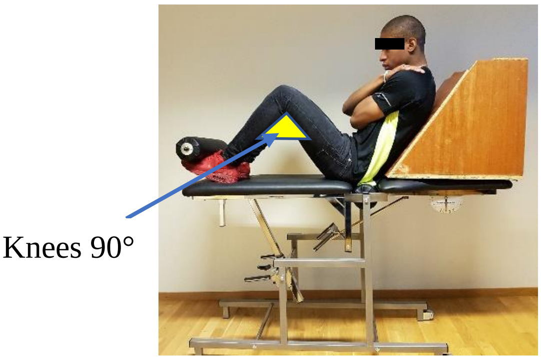

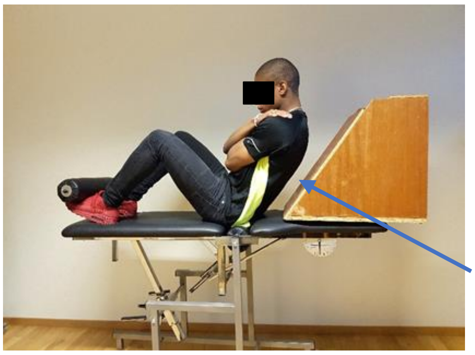

10 cm distance

Test 3, Side bridge on either side. Right side up first as a procedure.

Floor mat.

The candidate is placed sideways on the mat with straight knees and upper leg in front of the lower with toe against heel. Upper body is supported with elbow on the floor resting free hand supporting the weight-bearing opposite shoulder. The test starts once the body is in a straight line.

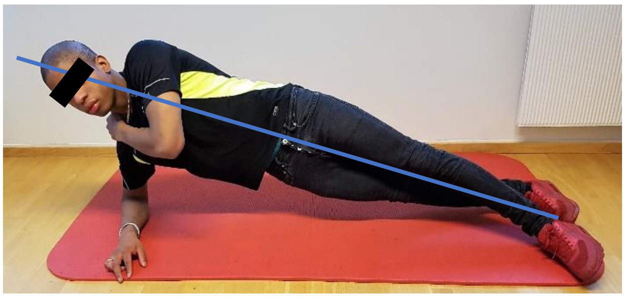

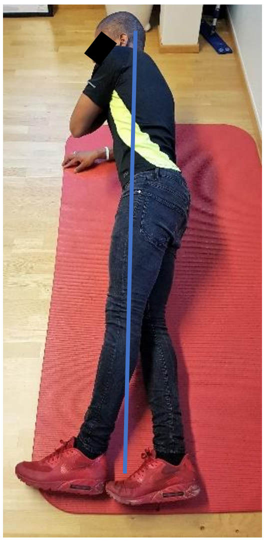

1. McGill S. Low back disorders: evidence-based prevention and rehabilitation: Human Kinetics; 2007.
