# Supplementary material for: Impact of exercise programs among helicopter pilots with transient LBP
Source: BMC Musculoskelet Disord. 2017 Jun 20;18:269. doi: 10.1186/s12891-017-1631-0 (PMC5477756; doi:10.1186/s12891-017-1631-0)
Supplement: Supplementary file 2 — Training program A. (6130 kb) [file 12891_2017_1631_MOESM2_ESM.pdf]

Workout plan Programme A

Helicopter pilots with transient low back pain.

All exercises: 3 series x 10 repetitions

Exercise	Illustration	Focus	Exercise data	Comments
1 - Arching - swaying, standing on all fours	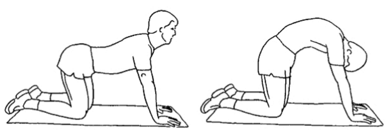	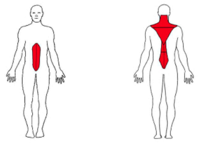	3 x 10	Start on all fours. Arch your back, looking down towards your navel and hold the position for 4-5 sec. Reverse the arch looking up and hold for 4-5 sec., then rest for 4-5 sec.
2 - "Bridge"	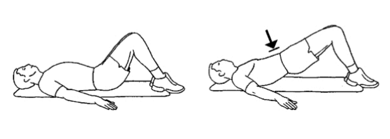	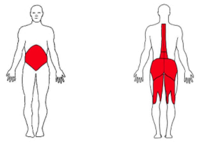	3 x 10	Lie on your back with the soles of your feet on the floor and your arms out to the side. Tilt your pelvis backward. Activate the lower part of your abdominal muscles, pull your navel inward and "flatten" your abdomen. Raise your pelvis from the floor until you lie on your shoulder blades only. Lower and raise slowly.
3 - Diagonal lift, standing on all fours	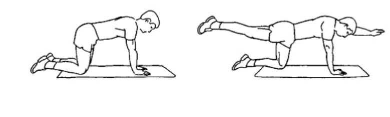	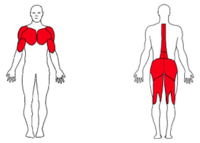	3 x 10	Start on all fours with your face facing the floor. Contract your abdomen and lower back for stability. Stretch one arm and the opposite leg to extend of your body. Repeat the movement with opposite arm and leg.
4 - Lying pelvis rotation 2	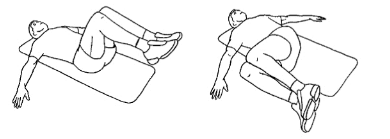	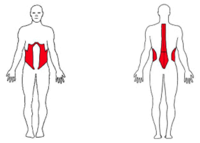	3 x 10	Lie on your back with your hips and knees bent 90 degrees and arms lying to the sides. Alternately rotate your pelvis and legs to your right and left. Keep your legs together.
5 - Diagonal lift, lying face down	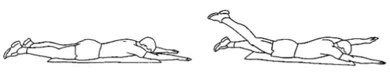	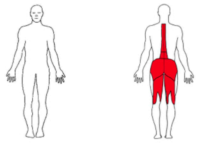	3 x 10	Lie flat, facing down with your arms straight above your head. Lift one arm and opposite leg, in stretched position, off the floor and slowly lower them again. Lift opposite arm and leg.
6 - Squat stability	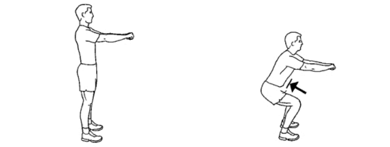	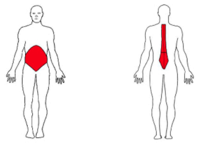	3 x 10	Stand erect and find the neutral position of your back and pelvis. "Flatten" your abdomen and bend your knees until in a seated position. Push back up and repeat.
7 - Pelvis lift w/knee stretching	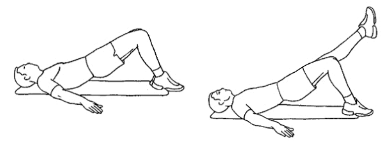	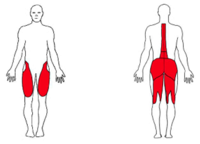	3 x 10	Lie on your back with bent knees and your feet on the floor. Contract your buttocks muscles and raise your pelvis and lower back. Hold the position and straighten one leg. Hold that position for 3-5 seconds and slowly lower back. Repeat with opposite leg.
8 - Crunch w/knee lift	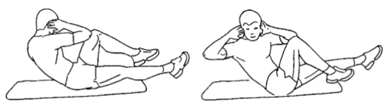	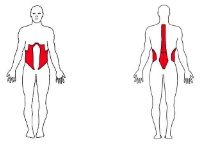	3 x 10	Sit on the floor with your hands behind your head. leaning backward, raise your legs slightly from the floor and find your balance. Alternately move opposite knee and elbow toward each other.
9 - The plank 1	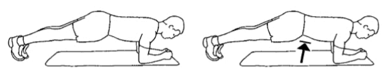	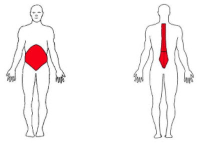	3 x 10	Stand on your elbows and the tips of your toes, find the neutral position of your back and pelvis. "Flatten" your abdomen, holding the position for 10-15 seconds.
10 - Side-lying side lift 1	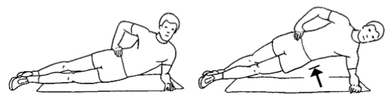	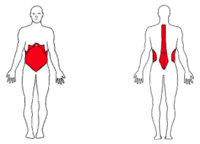	3 x 10, each side	Lie on your side supporting yourself on your elbow. Find the neutral position of your back and pelvis. "Flatten" your abdomen and raise your pelvis until your body is straight. Hold for 10-15 seconds and rest equally. Repeat to opposite side.
