# Supplementary material for: Impact of exercise programs among helicopter pilots with transient LBP
Source: BMC Musculoskelet Disord. 2017 Jun 20;18:269. doi: 10.1186/s12891-017-1631-0 (PMC5477756; doi:10.1186/s12891-017-1631-0)
Supplement: Supplementary file 3 — Training program B. (395 kb) [file 12891_2017_1631_MOESM3_ESM.docx]

**
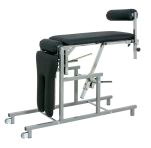
 Angle bench**

**Chest piece**

Stepless regulated

**Foot piece**

Stepwise regulated

**Foot support**

**General instructions**

Basic period over 36 training sessions not more frequently than every other day. 10 minute general warm up before start.

**1 Extension**

**Bench settings:** Foot piece; one click down (10°)

Chest piece; 41° in relation to foot piece

**Execution:** Lift upper torso in a steady fast tempo to a straight body line avoiding any extension, also of the cervical spine. Maintain an isometric straight body line for 6 seconds. Eccentric phase of at least 7 seconds returning to starting point. “Bracing” initiated and released between each repetition reducing the intensity of the bracing procedure with improved skill. Number of repetitions according to submaximal individual capacity in 4 series with increasing numbers according to reversed pyramid protocol of maximally 7-6-5-4 repetitions. At least 4 training sessions at any level before increasing by one repetition per series. One minute intermission involving brisk walking or biking between every series. Once achieved 7-6-5-4 repetitions the load is increased by applying weight west starting with 2 kg and increasing load by 1 kg every fourth training session.

**
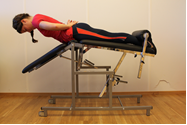

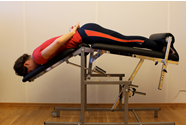
**

**2 “Curl-up”**

**Floor mat**

**
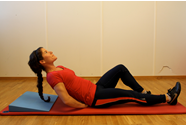

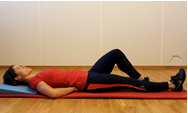
Execution:** Hand placed under the lumbar spine for support. Tongue firmly against the palate. One knee bent at 90° with sole of foot against the floor. Steady fast lifting the upper torso in a straight line avoiding “pulling-up” by head flexion to a low angle. Maintain an isometric position for 6 seconds. Eccentric phase of at least 7 seconds returning to starting point. “Bracing” initiated and released between each repetition reducing the intensity of the bracing procedure with improved skill. Number of repetitions according to submaximal individual capacity in 4 series with increasing numbers according to reversed pyramid protocol of maximally 7-6-5-4 repetitions. At least 4 training sessions at any level before increasing by one repetition per series. One minute rest on floor between series. Once achieved 7-6-5-4 repetitions the load is increased by applying weight west starting with 1 kg and increasing load by 1 kg every fourth training session.

**3 Bilateral leg extension**

**Bench settings:** Foot piece; one click down (10°)

Chest piece vertical

**Execution:** Prone position with chin and head resting on the hands. Raise both legs simultaneously in a steady fast tempo while gradually externally rotating both feet. Keep the legs (knees) fully extended at all times. The body should be in a straight line avoiding hyperextension. The overall body position on the angle bench must always be customized according to individual capacity as to seek the best possible working angle. Maintain an isometric position for 6 seconds. Eccentric phase of at least 7 seconds returning to starting point. “Bracing” initiated and released between each repetition reducing the intensity of the bracing procedure with improved skill. Number of repetitions according to submaximal individual capacity in 4 series with increasing numbers according to reversed pyramid protocol of maximally 7-6-5-4 repetitions. At least 4 training sessions at any level before increasing by one repetition per series. One minute intermission involving brisk walking or biking between every series. Once achieved 7-6-5-4 repetitions the load is increased by applying weights on both ankles starting with 1 kg during four sessions. Load is increased to a maximum of 2 kg to be continued throughout the remaining basic training program of 36 sessions.

**
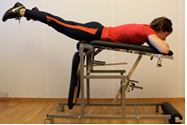

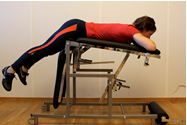
**

**4 Upper Torso Lateral Raise**

**Bench settings:** 1^st^ set foot piece to 25° (3 clicks down from level position).

2^nd^ adjust chest piece to appropriate angle according to individual capacity (ranging from +5°/ 0°{level} / ÷5° / ÷10 / ÷15° or ÷20°).

**Execution:** Sideways on the angle bench with lower leg bent 90° and securing the lower leg with a straight upper leg beneath foot support. Body in a straight line with feet, pelvis and shoulder centrally placed on the bench. The working angle of the chest piece is set according to individual capacity. Lift the torso sideways to a straight position in a steady fast tempo avoiding any lateral flexion of the cervical spine. Maintain an isometric position for 6 seconds. Eccentric phase of at least 7 seconds returning to starting point. “Bracing” initiated and released between each repetition reducing the intensity of the bracing procedure with improved skill. Number of repetitions according to submaximal individual capacity in 4 series with increasing numbers according to reversed pyramid protocol of maximally 7-6-5-4 repetitions. The exercises are performed consecutively on every other side series by series. Increasing load by increasing working angle in increments as suggested every fourth training session. After maximum working angle is achieved, further loading by weight west starting with 2 kg and adding 1 kg every fourth training session.

**
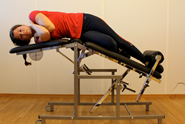

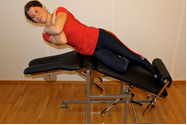
**

Protractor to guide the working angle
